# Impedance analysis of adherent cells after in situ electroporation-mediated delivery of bioactive proteins, DNA and nanoparticles in µL-volumes

**DOI:** 10.1038/s41598-020-78096-6

**Published:** 2020-12-07

**Authors:** Judith A. Stolwijk, Joachim Wegener

**Affiliations:** 1 Institut fuer Analytische Chemie, Chemo- & Biosensorik, Universität Regensburg, Universitaetsstr. 31, 93053 Regensburg, Germany; 2 Fraunhofer Einrichtung fuer Mikrosysteme und Festkörpertechnologien EMFT, Universitaetsstr. 31, 93053 Regensburg, Germany

**Keywords:** Biophysical methods, Gene delivery

## Abstract

Specific intracellular manipulation of animal cells is a persistent goal in experimental cell biology. Such manipulations allow precise and targeted interference with signaling cascades, metabolic pathways, or bi-molecular interactions for subsequent tracking of functional consequences. However, most biomolecules capable of molecular recognition are membrane impermeable. The ability to introduce these molecules into the cytoplasm and then to apply appropriate readouts to monitor the corresponding cell response could prove to be an important research tool. This study describes such an experimental approach combining in situ electroporation (ISE) as a means to efficiently deliver biomolecules to the cytoplasm with an impedance-based, time-resolved analysis of cell status using electric cell-substrate impedance sensing (ECIS). In this approach, gold-film electrodes, deposited on the bottom of regular culture dishes, are used for both electroporation and monitoring. The design of the electrode layout and measurement chamber allows working with sample volumes as small as 10 µL. A miniaturized setup for combined electroporation and impedance sensing (µISE-ECIS) was applied to load different adherent cells with bioactive macromolecules including enzymes, antibodies, nucleic acids and quantum dot nanoparticles. The cell response after loading the cytoplasm with RNase A or cytochrome c (in the presence or absence of caspase inhibitors) was tracked by non-invasive impedance readings in real-time.

## Introduction

The transfer of nucleic acids including DNA, RNA, and siRNA into living mammalian cells has evolved as an important tool across all fields of biomedical research as these molecules enable studying or specifically altering cellular functions^1^. Similarly, the delivery of proteins and peptides including antibodies and enzymes or enzyme substrates to interfere with cell function on the protein level constitutes a long-sought goal in cell biology as the application of these molecules is otherwise limited to extracellular targets or fixed and permeabilized tissues^2,3^. Moreover, a myriad of nanoparticle probes or sensors have been developed over the last decades that require precise targeting inside the cytoplasm for defined intracellular sensing^4^. Accordingly, there is a need for efficient delivery techniques that provide access to the cytoplasm for otherwise non-membrane permeable solutes.

Electroporation has proven to be a versatile technique that enables the introduction of different polar and high molecular weight molecules and probes directly into the cytoplasm of most cells by the application of short invasive electric pulses^5^. Technically, most electroporation is carried out upon cells in suspension, however in the studies to be presented, in situ electroporation (ISE) will be employed where cells are electrically pulsed while remaining attached to their growth substrate^6^. The procedure offers advantages over cell suspension electroporation as cell–cell and cell-substrate contacts are fully established and functional, which accelerates regeneration and thereby significantly increases survival rates. Moreover, ISE avoids enzymatic detachment of the cells from the growth surface and the associated cell trauma before pulsing. From the experimental perspective, in situ electroporation allows for an immediate post-pulse analysis of the cells with respect to structural or functional changes induced by the molecules delivered. After electroporation, the cells were typically examined by microscopic, biochemical, or molecular biological techniques. These are often end-point assays that may require additional labelling or staining. Alternatively, real-time techniques have been described, which are mainly based on video-microscopy reporting on the uptake of extracellular material or the release of cytoplasmic molecules^7^. In addition to imaging, electrochemical approaches have been used to monitor membrane integrity of single cells during pulsing^8^, the barrier function of cell layers before and after in situ electroporation^9^, the effect of suspension electroporation on post-pulse cell layer formation^10^ or the electrical properties of a tissue^11^. Whereas these techniques were essentially applied to monitor the invasiveness of an electric pulse, none of them was used to record the cell response to the presence of bioactive molecules that had been delivered to the cytoplasm by means of pulsing.

In previous research, we have combined in situ electroporation (ISE) with impedance-based monitoring of the cells before, during and after the electroporation pulse^12,13^. Electric Cell-Substrate Impedance Sensing (ECIS) provided real-time and label-free cell monitoring^14^. The cells were grown on coplanar gold-film electrodes on the bottom of a cell culture dish, and these electrodes were used for both in situ electroporation and concomitant impedance measurements to follow the cell response to pulsing and delivery^13^. In ECIS, the impedance of a cell-covered electrode is measured over time at designated AC frequencies. The recorded impedance monitors changes in cell shape, as the current (below certain threshold frequency) essentially flows around the insulating cell bodies^14^. Due to its sensitivity to morphological changes, a typical cell reaction to various kinds of external stimuli, ECIS is used to monitor the impact of the electroporation pulse itself as well as the response to the bioactive molecule delivered to the cytoplasm. For example, this ISE-ECIS technique has been applied to record the impact of the membrane-impermeable anti-cancer drug bleomycin after its ISE-mediated delivery into normal rat kidney (NRK) cells^12^.

In the past, the dimensions of the conventional ECIS electrode layout, using a small working electrode with a large counter electrode (type 8W1E, Applied BioPhysics Inc. (USA)), limited the minimum sample volume to ~ 150 µL. Since the diffusion of exogenous molecules across the plasma membrane is only possible *during* the brief electroporation pulse, it is necessary to have high extracellular concentrations for efficient cell loading. Unfortunately, for many bioactive molecules of interest such as antibodies or nucleic acids, it is not practical to obtain the large quantities required. One approach to increase the effective concentration with relatively small quantities of these molecules of interest is to reduce the sample volume during electroporation. Such volume reduction, however, can be a challenge for which different approaches have been proposed by other groups^15–19^.

In the research described in this paper, we have made ‘small volume ISE’ compatible with concomitant impedance monitoring of the cells. To accomplish this, a new electrode setup was established which uses two small gold-film electrodes of the same geometry separated by just a few hundred micrometers. These electrodes are contained within a small silicone chamber, holding a total volume of 50 µL. The electrode pairs were patterned to provide an 8-well array for parallel experiments and connected to an impedance analyzer or pulse generator through computer-controlled relays. The entire setup allows one to perform ISE-ECIS experiments in a total volume of only 10 µL. We refer to this novel arrangement as µISE-ECIS.

The established µISE-ECIS setup demonstrates the ability to monitor the cytoplasmic activity of selected bioactive proteins. Cells were electroporated in the presence of cytochrome c that triggers apoptosis when present in the cytoplasm and the enzyme RNase A that cleaves single-stranded RNA. The response of the cells to these proteins was followed by changes in impedance, which reflect changes in cell electrode coverage or more subtle morphology changes within the cell layer. Moreover, we demonstrate the µISE-mediated loading of cells with antibodies and DNA molecules. To set the stage for intracellular applications of nanoparticles with therapeutic or analytical functions, we successfully delivered fluorescent quantum dot nanoparticles in cells utilizing the µISE-ECIS approach.

## Methods

### Cell culture

Cell lines NRK-52E, HEP-G2, CHO-K1 and HEK-293 were purchased from the German Collection of Microorganisms and Cell Cultures DSMZ (www.dsmz.de). NRK-52E and HEK293 cells were cultured in Dulbecco’s modified Eagle’s medium with 4.5 g/L d-glucose (Sigma-Aldrich) supplemented with 10% (v/v) fetal calf serum (Biochrom), 100 µg/ml penicillin/streptomycin (Sigma-Aldrich) and 2 mM L-glutamine (Sigma-Aldrich). For HEP-G2 cells RPMI-1640 medium (Sigma-Aldrich) was supplemented with 10% (v/v) fetal calf serum, 100 µg/ml penicillin/streptomycin and 2 mM L-glutamine. CHO cells were cultured in Alpha-Medium (modified MEM) (Sigma-Aldrich) with 10% (v/v) fetal calf serum, 100 µg/ml penicillin/streptomycin and 2 mM L-glutamine. Cells were kept in ordinary humidified cell culture incubators at 37 °C with 5% CO_2_. The culture medium was changed twice a week. All routine sub-culturing was performed by standard trypsinization protocols using 0.25% (w/v) trypsin plus 1 mM EDTA in PBS (Sigma-Aldrich).

For experiments with HEK293 cells, the gold-film electrodes (see below) were pre-coated by using 0.5% (w/v) gelatine (Sigma-Aldrich) in phosphate-buffered saline (PBS) for 2 h at room temperature. The gelatine layer was subsequently cross-linked with 2.5% (w/v) glutaraldehyde (Merck) for 10 min. Excess glutaraldehyde was removed by washing the substrates thoroughly (10 times) with deionized water. Cell layers were grown to confluence and routinely inspected by phase contrast microscopy prior to any ISE-ECIS experiment.

### Experimental setup

**Figure 1:**
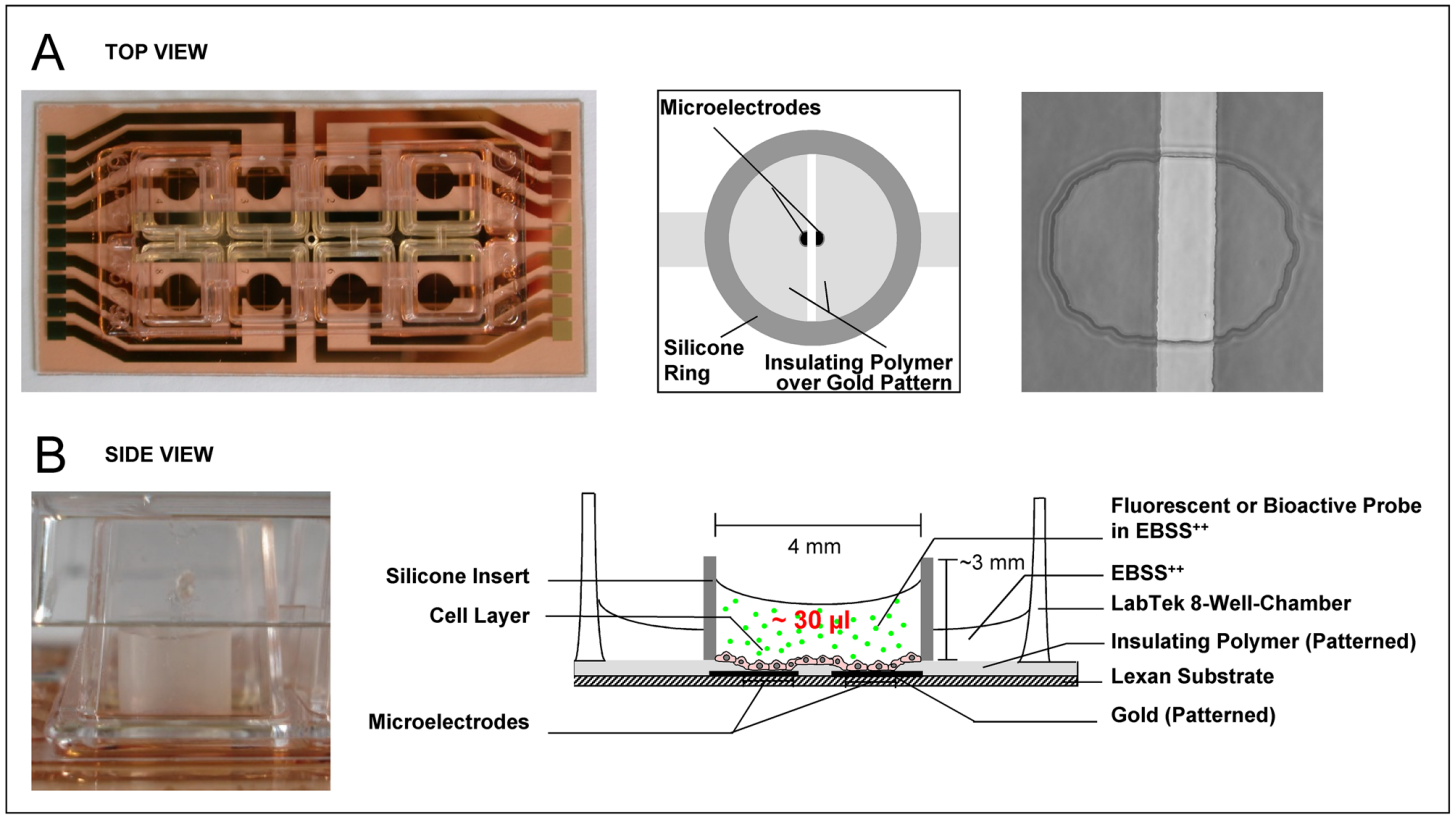
Experimental setup for combined in situ electroporation (ISE) and impedance-based cell monitoring (ECIS) in small sample volumes. (**A**) Top view. (**B**) Side view. (**A**) Top view of an 8-well electrode array (left), a schematic top view into one well of the electrode array after modification with an inserted silicone ring (middle) and a phase contrast micrograph of the two semi-circular, cell-free electrodes defined by openings in an overlaying passivation layer (right). (**B**) Side view of one well of an electrode array modified with a silicone chamber (left) and schematic side view into one well (right). Note that dimensions are not to scale. Details are given in the main text.

Impedance-based monitoring of adherent cells before and after electroporation was performed using the well-established ECIS technology^12,13,20^. The small volume measurement chambers consist of a custom-made 8-well cell culture dish with gold film electrodes deposited on the bottom of each well (Applied BioPhysics Inc.) (Fig. 1A).

All wells of these 8 well electrode arrays contain two half-circular electrodes, each with a surface area of about 2.5 × 10^–4^ cm^2^ separated by a gap of 100 µm (cf. Fig. 1A). The active electrodes are defined by corresponding openings in a biocompatible passivation layer, insulating the most of the gold film against the electrolyte. Each of the 16 electrodes is connected by gold traces to a contact area at the periphery of the *Lexan* base substrate. Due to geometrical constraints, the contact points are equally split to the two opposing sides of the array. Data of four wells has been collected during a single experiment. To create the wells for small volume applications (10–30 µL) a silicone ring with an inner diameter of 4 mm and a height of about 3 mm was affixed to the bottom of the well using a non-toxic silicone adhesive (Warenimport und Handels GmbH, Vienna). The silicon ring was cut from meter ware silicon tubes (VWR). After 24 h of silicone curing, the arrays were sterilized in an argon plasma cleaner (Harrick Plasma) for 15 s. For electroporation experiments, the molecules to be delivered were added to the cell population inside the silicone chamber (Fig. 1B). In order to prevent critical solvent evaporation from these small reservoirs, sacrificial buffer (100–200 µL) was added to the space surrounding the inner chamber. The 8well chambers were capped with a polystyrene lid during the entire course of an experiment.

The 8well electrode array was placed in a humidified cell culture incubator at 37 °C with 5% CO_2_ and interfaced to the electronic equipment located outside of the incubator. For µISE-ECIS experiments, we used either the all-in-one instrument ECIS 1600R (Applied BioPhysics Inc.) or an equivalent setup comprising a microcontroller, a relay, an impedance/gain-phase analyzer (SI-1260, Solatron Group Ltd.) and a pulse generator (Agilent 33120A, Agilent Technologies) as described previously^12,13^.

Impedance data of the cell-covered electrodes were recorded at an AC frequency of 4 kHz^12^. Due to the same size of the two electrodes, the readout provides the total impedance from both cell-covered electrodes in series. To apply an electroporation pulse at a given time during ECIS data acquisition, the measurement was paused and the electrodes were switched to the pulse generator by computer controlled relays. In situ electroporation was achieved using sinusoidal voltages at an AC frequency of 40 kHz^12^ using pulse amplitudes (in V_rms_) and durations (in ms) depending upon cell type: NRK-52E: 5 V/200 ms; HEK293: 7 V/200 ms; HEP-G2: 5 V/200 ms; CHO: 3 V/500 ms. The impedance magnitude |Z| at any time of the measurement was normalized to the last |Z| value before the electroporation pulse, denoted as |Z|_E_. Since the voltage drops equally across both electrodes in first approximation (as long as the impedance of both electrodes is the same) the cell population on both electrodes will be electroporated.

### Electroporation of fluorescent dyes

A membrane-impermeable fluorescent dye was used to assess the loading efficiency during electroporation-mediated delivery. FITC-dextran (250 kDa, Sigma-Aldrich) was dissolved in Earle’s balanced salt solution supplemented with calcium and magnesium (EBSS^++^; Sigma-Aldrich) in a concentration of 2 mg/ml. After the addition of the probe solution, cells were re-equilibrated to incubator conditions for at least 30 min, which was followed by online ECIS readings before the electroporation pulse was applied. Cells were left in the incubator for an additional 15 min after the pulse and were then washed three times with EBSS^++^. Dye uptake was documented using an upright confocal fluorescence microscope with a 10 and 63× water immersion objective (see below).

### Electroporation of bioactive proteins and enzymes

Cytochrome c from equine heart (Sigma-Aldrich) was used at a concentration of 10 mg/ml in EBSS^++^. Caspase inhibitor Ac-AEVD-CHO (Sigma-Aldrich) was used in co-electroporation experiments at a concentration of 200 µM. Extracellular RNase A (Roche Diagnostics) concentrations were 0.1, 0.25 and 0.5 mg/ml. Heat inactivation of RNAse for control conditions was performed by boiling the enzyme at 100 °C for 30 min. After electroporation, the cell response was monitored by ECIS readings for at least two hours. At the end of an µISE-ECIS experiment, the nuclei of the cells were stained using 10 ng/ml DAPI (Sigma-Aldrich) in PBS^++^ for 10 min.

### Electroporation of antibodies

To establish the in situ electroporation of antibodies, we selected rabbit-anti-ß-catenin IgG (Sigma-Aldrich) at a final concentration of 2.8 mg/ml, and rabbit-anti-occludin IgG (Zymed Laboratories) at a working concentration of 125 µg/ml as well as the secondary antibody Alexa Fluor 568-labelled goat anti-mouse secondary antibody (Molecular Probes) at a concentration of 1 mg/ml in EBSS^++^.

Following µISE-mediated loading with primary antibodies (anti-ß-catenin, anti-occludin), cells were washed, fixed with 4% paraformaldehyde (v/w; Merck), permeabilized with 0.2% (v/v) Triton-X 100 (Sigma-Aldrich) and unspecific binding was blocked with 3% (w/v) bovine serum albumin (Sigma-Aldrich) in PBS^++^. Then, cells were stained with the secondary antibody Alexa Fluor 488-labelled goat anti-rabbit (Molecular Probes). Antibody uptake was assessed by confocal fluorescence microscopy using an upright fluorescence microscope with a 63× water immersion objective. After ISE of cells in the presence of the Alexa Fluor 568-labeled goat secondary antibody cell layers were washed twice with EBSS^++^ before live-cell fluorescence microscopy was performed.

### Electrotransfection

For electroporation-mediated DNA transfections of NRK, Hep-G2 and HEK 293 cells we used the plasmid (1) pcDNA 3 (Invitrogen) carrying the EGFP gene insert from pEGFP-N1 (Clontech) or (2) the pEGFP-Actin plasmid (Clontech) at a concentration of 180 µg/ml and a total volume of 30 µL EBSS^++^. About 15 min after electroporation, 200 µL full culture medium was added to the cell layers. Cells were allowed to synthesize protein from the DNA template for 24 h before the transfection efficiency was evaluated by confocal fluorescence microscopy using an upright fluorescence microscope with a 10× water immersion objective. Transfection efficiency was evaluated in a semi-quantitative way by reporting the fraction of GFP/YFP-positive cells compared to the total cell number.

### Electroporation of quantum dots

PEGylated *QTracker* quantum dots (Q21031MP, Invitrogen) were diluted in EBSS^++^ to a final concentration of 0.4 µM. About 20–30 min after electroporation, cell layers were washed three times with EBSS^++^ and subsequently prepared for inverse epifluorescence microscopy (Leica: ex 340–380 nm/em 425 nm or Nikon: ex 330–380 nm/em 420 nm).

### Fluorescence microscopy

To image cells after electroporation by fluorescence microscopy, the 8well chambers of the electrode array was removed. The cells were studied using either an inverted microscope equipped with an epifluorescence illumination unit (Nikon Diaphot, Japan) or upright microscopes (Nikon Eclipse 90i, Japan, or LEICA TCS SL, Leica, Germany) in wide-field or confocal laser scanning microscopy (CLSM) mode. For imaging using inverted microscopes, the cells on the array were embedded in Aqua PolyMount (Polysciences Inc.), covered with a cover glass, and flipped upside down before microscopy with a PLAN 20×/0.4 objective. In upright microscopes water-immersion objectives (Nikon: NIR Apo 60×/1.0 W, Leica: HCX APO L 10×/0.3 W, 10×, HCX APO L 63×/0.9 W) were dipped into the pre-warmed buffer overlaying the cell-covered electrodes. Embedded samples were inspected using a HCX APO L 63×/0.9 W objective on the LEICA TCS SL.

## Results and discussion

### Characterization of the µL-volume ISE-ECIS setup

**Figure 2:**
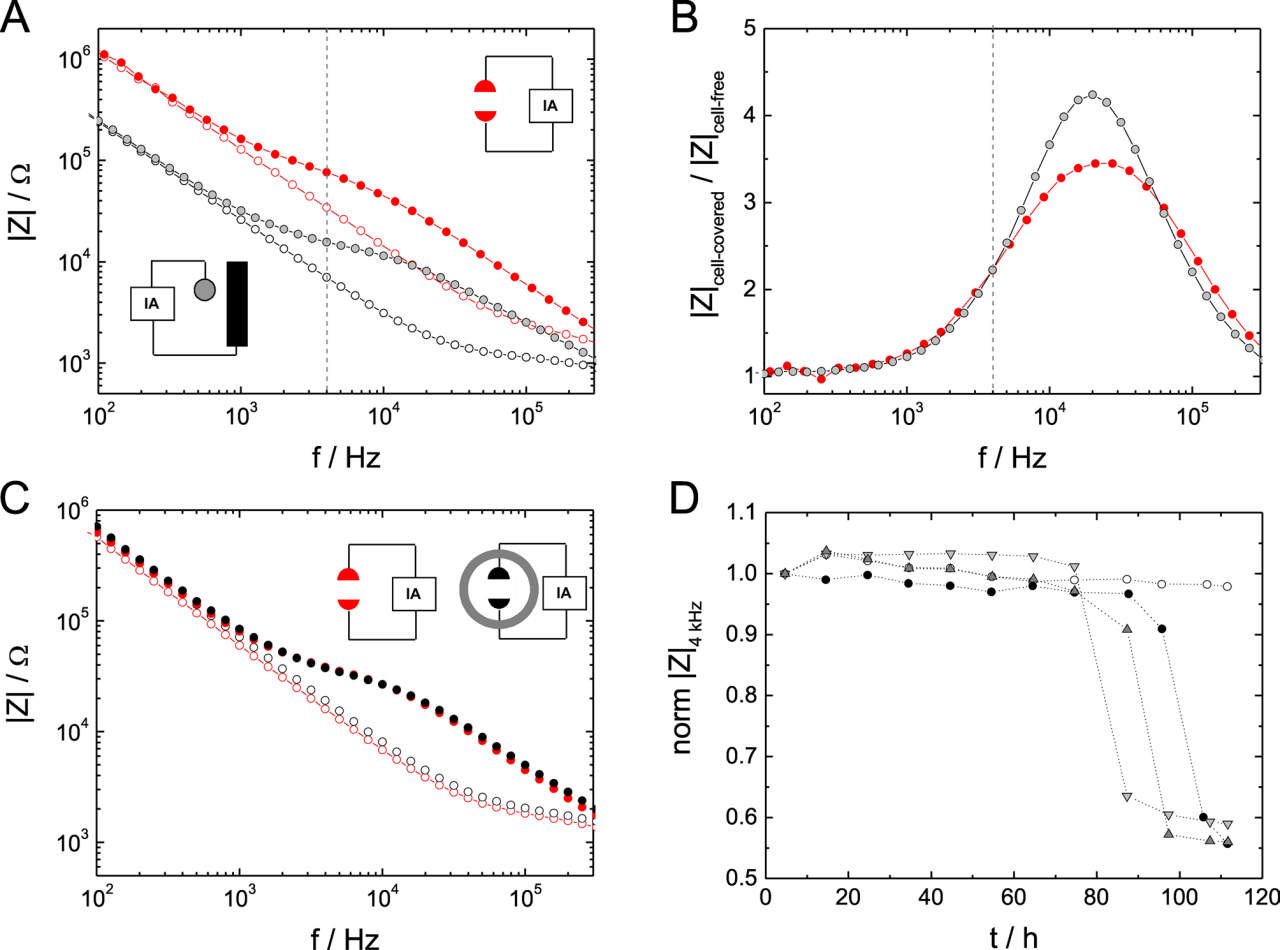
Electrochemical characterization of the micro-electrode array for µISE-ELPO. (**A**) Impedance spectra for cell-free electrodes (open symbols) are compared to those recorded for the same electrodes covered with a confluent layer of NRK cells (filled symbols): Equally-sized micro-electrodes for µISE-ECIS (2.5 × 10^−4^ cm^2^ each, red) versus the common small working electrode (5 × 10^−4^ cm^2^)/big counter electrode arrangement (grey). Pictograms illustrate the respective electrode configurations. (**B**) The corresponding normalized impedance spectra (|*Z*|_cell-covered_/|*Z*|_cell-free_). (**C**) Impedance spectra for cell-free and cell-covered micro-electrodes before (red) and after (black) modification with a silicone ring. (**D**) Time course of the normalized impedance (|*Z*|_t_ /|*Z*|_t0_) for NRK cell layers grown on microelectrodes loaded with different volumes of EBSS^++^ buffer (10 µL—inverted triangles, 20 µL—triangles, 30 µL–filled circles) compared to a control volume of 400 µL (open circles).

We first characterized the modified microelectrode-arrays for combined in situ electroporation and ECIS in µL-volumes (µISE-ECIS) in terms of electrical characteristics and performance. The custom-made microelectrode arrays contain two small (~ 2.5 × 10^−4^ cm^2^) half-circular gold-film electrodes per well separated by 100 µm (cf. Fig. 1). Dependent on the mask alignment during photolithography, the two opposing microelectrodes ideally have an identical surface area and are connected in series to each other via the bulk electrolyte. Thus, both electrodes contribute equally to the total impedance of the system as long as the cell layer is homogeneous with respect to its dielectric structure. This situation is different from conventional micro-electrode layouts with one small working electrode and a much bigger counter electrode, e.g., 8W1E from Applied BioPhysics. In such an electrode layout, the cell-covered working electrode dominates the overall impedance, and the counter electrode is masked^14^. Figure 2A compares the frequency-dependent impedance magnitude |Z| of the electrode layout used here for µISE-ECIS (red) to an electrode layout with one working electrode of the same total surface area (2 times 2.5 × 10^−4^ cm^2^ = 5 × 10^−4^ cm^2^) in combination with a much bigger counter electrode (black). Due to the reduced surface area of the two individual micro-electrodes in µISE-ECIS and their serial arrangement (impedances are added), their impedance spectra are shifted to higher impedance values (Fig. 2A) compared to one micro-electrode of the same total size in combination with a much bigger working electrode. From impedance theory, one would expect a fourfold increase in impedance for the µISE-ECIS electrodes, which is experimentally verified (cf. Fig. 2A). For both electrode types, the influence of the cell layer is clearly measurable in a frequency range between 1 to 500 kHz. The contribution of the cell layer to the overall impedance magnitude is most clearly highlighted by plotting the *normalized* impedance, which is the ratio of the impedance of the cell-covered and the cell-free electrode (|Z|_cell-covered_/|Z|_cell-free_) for each individual frequency (Fig. 2B). Values greater than one indicate corresponding impedance contributions by the cell layer relative to electrode and medium.

When voltage pulses are used for electroporation, the total voltage is applied to the serial arrangement of the cell layer, electrode and medium. Only at frequencies close to the maximum of the normalized impedance is the largest fraction of the applied voltage delivered to the cell layer rather than to the electrode interface or the medium. Thus, Fig. 2B provides the rationale that we used AC voltages of 40 kHz for pulsing, as reported earlier^9,10^.

The minimum buffer volume required for the micro-electrode arrangement was reduced by inserting a small silicone ring around the micro-electrodes. Any influence of the silicone insert on the electrical measurement was experimentally excluded (Fig. 2C), as the impedance spectra generated from electrodes either with or without the silicone ring overlapped for the entire frequency range. When working with small volumes, uncontrolled fluid evaporation is a major concern as it may increase the osmolality of the medium. Microelectrodes proved to be well-suited for long-term measurements in sample volumes of 10–30 µL for at least ~ 70 h without the cells being affected by evaporation effects (Fig. 2D). Beyond 70 h, the cell layer impedance collapsed in measurements with sample volumes of 10–30 µL. Although working in a sample volume of only 10 µL appeared justified for short-term measurements, we chose a sample volume of 30 µL in most experiments presented in this work. To date, there are only a few in situ electroporation setups known whose volume requirements are below 20 µL^21^, and most in situ electroporation setups require volumes between 80 µL and 500 µL^16,22^. These methods are typically applied to much larger cell populations in order to be compatible with established methods for later cell analysis.

**Figure 3:**
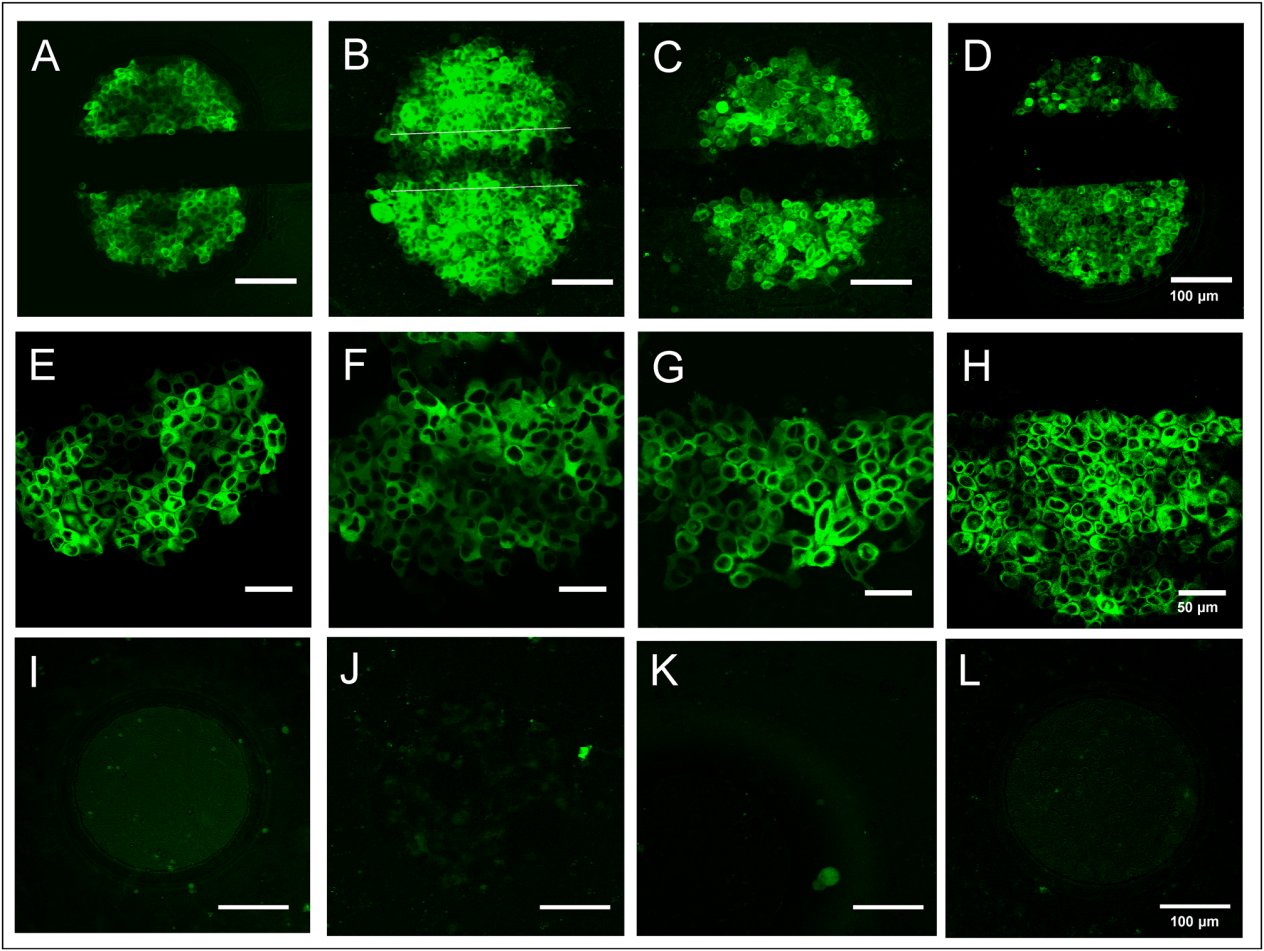
Confocal fluorescent micrographs of different adherent cell lines grown on micro-electrodes developed for µISE-ECIS after electroporation in the presence of 250 kDa FITC-dextran (2 mg/ml). (**A**, **E**) NRK; (**B**, **F**) HEK 293; (**C**, **G**) HEP-G2; (**D**, **H**) CHO. (**A**–**D**) Overview micrographs showing both cell-covered electrodes were taken with a 10× objective. (**E**–**H**) Magnified field of views from one half-circular microelectrode shown in **A**–**D** were taken with a 63× objective. **I** (NRK), **J** (HEK293), **K** (HEP-G2) and **L** (CHO) show monolayers of the different cell lines after incubation with the 250 kDa FITC-dextran (2 mg/ml) without any electroporation.

The micro-electrode setup was tested and optimized by µISE-mediated delivery of fluorescent dyes. These studies were intended to assess the electroporation efficiency. Confluent cell layers were pulsed using voltages of 40 kHz AC frequency with predefined sets of pulse amplitudes and durations (2–7 V for 200 ms or 500 ms) in EBSS^++^ supplemented with 2 mg/mL of a 250 kDa FITC-dextran as the fluorescent probe. Figure 3 shows confluent layers of NRK, HEK 293, Hep-G2 and CHO cells on micro-electrodes after in situ electroporation. All cells were successfully loaded with the FITC-dextran probe as indicated by the bright green cytoplasmic fluorescence of all cells residing on the electrode (Fig. 3A–D). Cells in the periphery of the electrodes do not show any sign of dye uptake, as they have not been exposed to the permeabilizing electric field. This finding demonstrates that there is no probe uptake in absence of the electric field, for instance, by leakage across the membrane or endocytosis. This conclusion is supported by Fig. 3I–L showing the four different cell types after incubation with the FITC dextran probe but without electroporation. There is no sign of probe uptake without pulsing. Figure 3E–H provide magnified images of the loaded cells. Fluorescence is confined to the cytoplasm and excluded from the nucleus as the 250 kDa probe is too big to diffuse through the nuclear pore complex. Dye loading of NRK, Hep-G2 and CHO cell layers is precisely restricted to the half-circular gold-film electrodes (Fig. 3A, C, D) excluding the 100 µm wide spacing between them. Dye uptake into HEK 293 cells (Fig. 3B), on the other hand, noticeably overlaps the electrode borders. This may be due to the multi-layered structure of confluent HEK 293 cells. Apparently, the electrical field that is constricted by the electrode borders spreads between the two opposing electrodes, and thereby partially permeabilizes the cells in between.

The pulse parameters for best dye uptake efficiency were, to some degree, cell type dependent (all 40 kHz): NRK (5 V/200 ms), Hep-G2 (5 V/200 ms), HEK 293 (7 V/200 ms) and CHO cells (3 V, 500 ms). Comparing these optimum pulse parameters to our previous studies^12^, we found the voltage amplitudes to be slightly elevated by approximately 1 V. This is obviously caused by the serial arrangement of two small, cell-covered electrodes which reduces the voltage drop across either one of them to about 50% of the totally applied voltage. In previous studies, we used one small working electrode and a much larger counter electrode. The latter contributed very little to the overall impedance. Thus, during pulsing, the voltage dropped almost exclusively across the small electrode and became very close to the total voltage applied. Nevertheless, the revised electrode geometry did not significantly increase the pulse amplitude required for efficient electroporation for all cells. CHO cells, for example, were successfully loaded with 3 V and 500 ms using both electrode layouts. This finding indicates that cell layer composition (cell size, cell layer thickness, cell layer impedance) also influences the voltage amplitude required for efficient electroporation, as these parameters determine the fractional voltage drop across the cell membranes. In first approximation, the voltage amplitude needed for successful electroporation scales inversely with the impedance contribution of the cell layer. The lower the impedance of the cell layer, the higher the threshold voltage needed. Membrane permeabilization requires app. an electric field strength of 1 kV/cm. The threshold amplitude also depends to a minor degree on how the impedance is distributed to trans- or paracellular current pathways across the cell layer which adds further evidence for the cell-type dependent electroporation voltages.

### Loading of adherent cells with Quantum Dot nanoparticles

Fluorescent particles in the nanometer size range have gained attention as passive labels for macromolecules or as active probes reporting on pH, oxygen, or temperature. Due to their small size (~ 1–10 nm) and extraordinary photo-physical properties, quantum dot nanoparticles have been used extensively for long term tracking or labeling of viable cells^23^. The use of nanoparticle sensors or drug delivery vehicles holds great promises for the future^24,25^. To date, most delivery approaches rely upon spontaneous, membrane-mediated cellular uptake mechanisms providing intracellular nanoparticles within endocytotic vesicles but without direct access to the cytoplasm. Only for ultra-small nanoparticles, spontaneous diffusion across the bilayer membrane into the cytoplasm has been observed. Thus, it takes delivery techniques that physically break the membrane barrier, such as electroporation or microinjection, to bring free particles into the cytoplasm where they can interact with different intracellular structures and target molecules^26^. However, it has been observed that QDs (quantum dots) tend to aggregate within the cytoplasm once they have been delivered by suspension electroporation. To date, monodisperse intracellular distribution of QDs has only been achieved when microinjection has been applied for delivery^26^.

**Figure 4:**
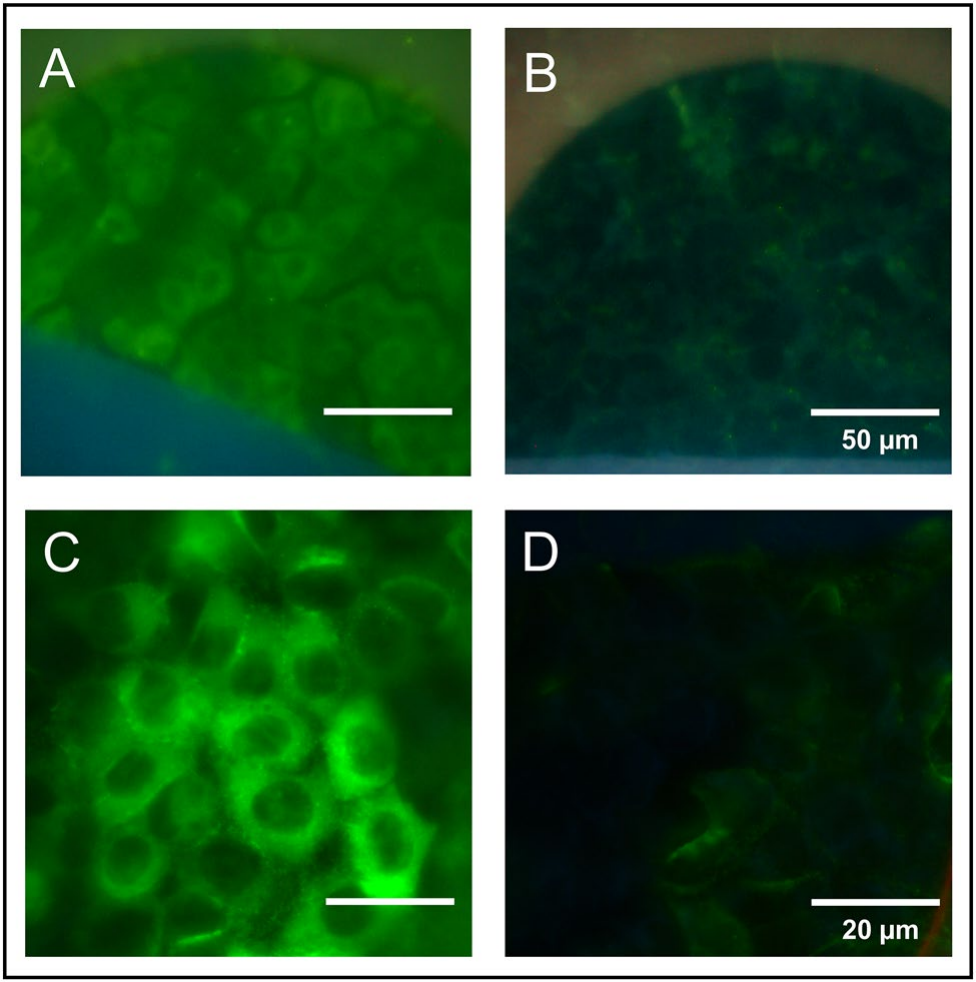
Electroporation-mediated loading of adherent cells with PEG-coated CdSe/ZnS core shell quantum dots (QD) nanoparticles. (**A**,**C**) Wide-field fluorescence micrographs of NRK cells grown on micro-electrodes after electroporation in the presence of 0.4 µM QDs. (**B**,**D**) Control cell layers were subjected to the quantum dot solution but were not permeabilized by electric pulsing. (**A**,**B**) show one half-circular micro-electrode covered with cells. (**C**,**D**) magnified regions from the center of both micro-electrodes, respectively. Electroporation was performed with 200 ms electrical pulses of 5.0 V amplitude at an AC frequency of 40 kHz.

The suitability of in situ electroporation for loading adherent cells with nanoparticles was tested using PEG-coated CdSe/ZnS core–shell quantum dots. The PEG-coating avoids adsorption of the particles to the cell membrane and thereby reduces unspecific endocytotic uptake. Figure 4 shows epifluorescence micrographs of NRK cells grown on micro-electrodes after electroporation in the presence of these quantum dot nanoparticles in a concentration of 0.4 µM (Fig. 4A, C). The QD particles fill the entire cytoplasm but remain excluded from the nucleus as their hydrodynamic diameter of ~ 23 nm^27^ is too big to pass the nuclear pore complex. The fraction of loaded cells was similar to experiments using 250 kDa FITC-dextran. The rather homogeneous distribution of the QDs within the cytoplasm indicates that they can diffuse freely and are not encapsulated in vesicles (Fig. 4C). Cell layers that were exposed to the same concentration of QDs without any electroporation (Fig. 4B, D) did not show any significant particle uptake within the time frame of the experiment (~ 30 min). Thus, loading of the cytoplasm of NRK cells adherently grown on the micro-electrodes is exclusively assigned to membrane permeabilization by electroporation. To our knowledge, the experiments presented here may be the first reports showing successful electroporation-assisted delivery of QDs into adherent cells in situ under physiological conditions.

### Electroporation-mediated loading with antibodies

Electroporation-mediated loading of viable cells with antibodies offers a very versatile spectrum of biochemical applications. Antibodies combine a consistent basic molecular structure with highly variable antigen-binding sites. Their enormous capability for molecular recognition may serve as a potent tool to specifically address and block intracellular functions, provided the antibodies obtain access to the cytoplasm of living cells. Combining in situ electroporation with impedance-based monitoring of the cell response may pave the way to precisely block a certain intracellular structure or interaction with highest specificity and to follow the associated cell response in terms of cell morphology, cell junctions or cell body dynamics.

**Figure 5:**
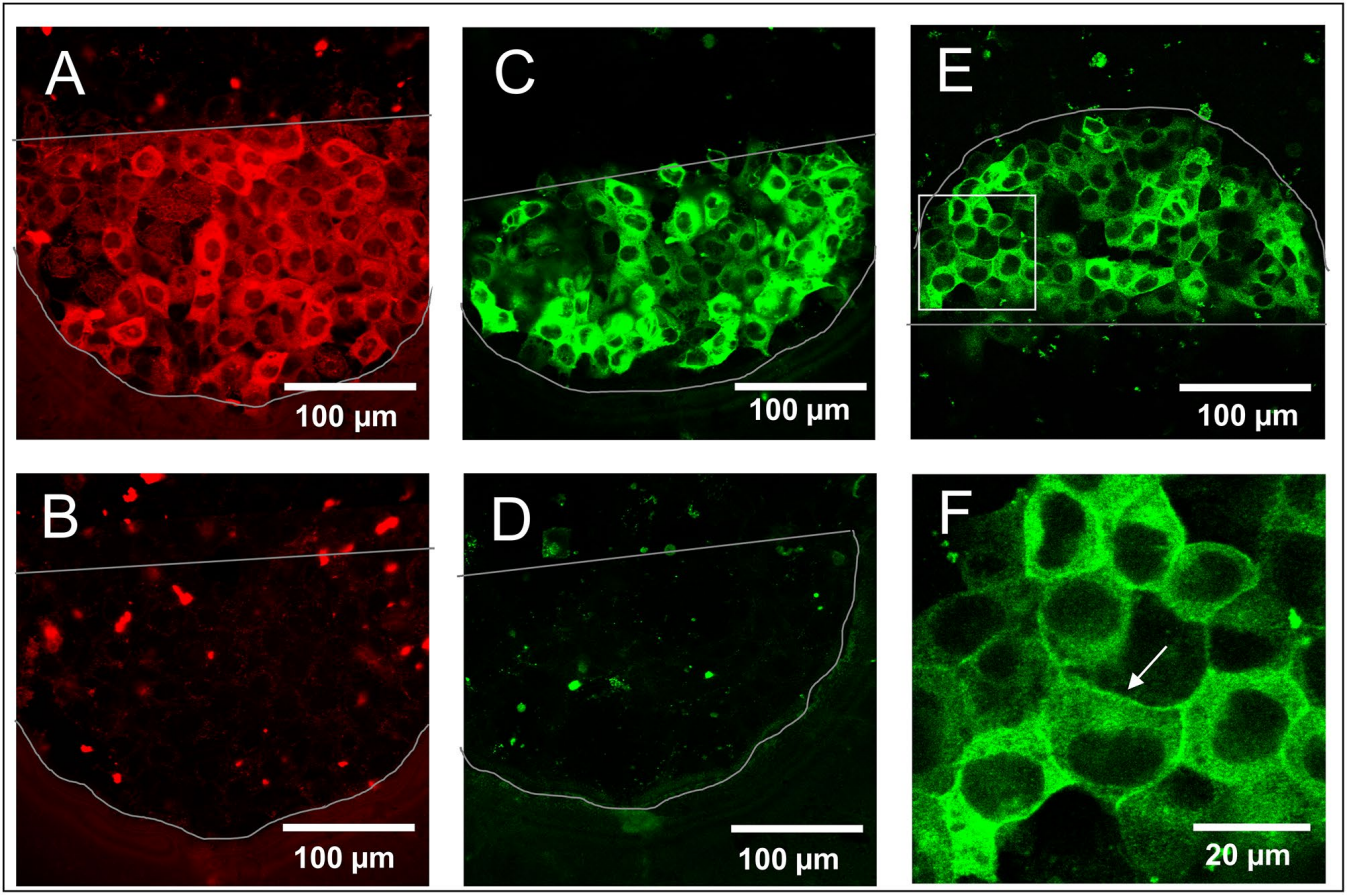
µISE-mediated loading of NRK cells with antibodies. Confocal fluorescence micrographs of NRK cells grown on half-circular electrodes (marked by grey outlines) after (**A**) electroporation in the presence of Alexa Fluor 546-labeled secondary antibody; (**B**) incubation with Alexa Fluor 546-labeled secondary antibody without electroporation; (**C**) electroporation in the presence of an anti-ß-catenin antibody followed by fixation, permeabilization and labelling of the bound anti ß-catenin antibody with an Alexa Fluor 488-labeled secondary antibody; (**D**) incubation with anti-ß-catenin antibody without electroporation followed by the same work-up as in (**C**); (**E**) electroporation in the presence of an anti-occludin antibody targeting the intracellular C-terminus followed by fixation, permeabilization and labelling of the bound primary antibody with an Alexa Fluor 488-labeled secondary antibody. (**F**) Magnification of a region of interest as outlined in (**E**) showing the localization of the anti-occludin antibodies at the cell–cell junctions (arrow). Electroporation was performed with 200 ms electrical pulses of 5.0 V amplitude at an AC frequency of 40 kHz.

As a first proof of principle, we studied the efficiency of electroporation-mediated antibody delivery to the cytoplasm using a fluorophore-labelled antibody that was not specific to any intracellular target. Figure 5A shows confluent NRK cells grown on gold-film microelectrodes after they had been exposed to an electroporation pulse in the presence of an Alexa Fluor 546-labeled secondary antibody. As demonstrated in the figure, cells grown on the electrode have become loaded with the labelled antibody. The fluorescence pattern indicates the presence of antibodies throughout the entire cytoplasm, whereas the nucleus remains dark, as the nuclear pore complex excludes diffusion of the antibodies into the nucleus. When NRK cells were incubated with this fluorescence-labelled secondary antibody, but not exposed to electroporation pulses, only some unspecific, punctate fluorescence was observed, presumably arising from extracellular antibody aggregates bound to the cell surface (Fig. 5B).

In another series of experiments, we used two different non-labelled primary antibodies recognizing individual intracellular target proteins associated with cell–cell junctions (Fig. 5C–F). Anti-ß-catenin specifically binds to ß-catenin, which connects the cadherin transmembrane proteins to the actin cytoskeleton. Anti-occludin recognizes the intracellular C-terminus of occludin, a transmembrane building block of the tight junction complex. After electroporation-mediated loading of NRK cells with non-labelled primary antibodies, these intracellular antibodies were visualized using well-established immuno-cytochemical staining protocols. The loaded cells were chemically fixed, permeabilized by detergents and exposed to fluorescence-labelled secondary antibodies that recognized the constant regions of the first antibodies. As shown in Fig. 5C,E, the secondary antibody stain confirmed the efficient loading of NRK cells with the anti-ß-catenin and anti-occludin antibodies, respectively. In contrast, a control cell layer (Fig. 5D), which was also incubated with the anti-ß-catenin antibody but not exposed to the electroporation pulse, does not show any significant intracellular staining. The latter finding confirms that membrane electroporation was responsible for the intracellular delivery of the antibodies to the cytoplasm. The experimental protocol, however, has an obvious disadvantage. Those primary antibodies that were delivered to the cytoplasm, but did not find their intracellular targets, will get irreversibly immobilized by the chemical fixation prior to incubation with the labelled secondary antibody. When particularly high extracellular antibody concentrations are used to ensure efficient loading, this staining protocol detects a rather high amount of primary antibodies that are not bound to their target but remain within the cells. This situation applies for the anti-ß-catenin labelling in Fig. 5C as the antibody was applied in significantly higher concentrations (2.8 mg/ml) as typically used by others^28,29^. Hardly any labelling of the cell borders is visible as it is masked by the unbound, yet immobilized primary antibodies (Fig. 5C). The anti-occludin-antibody (Fig. 5E) was applied at about a 20- fold lower concentrations (125 µg/ml) compared to anti-ß-catenin. Here the anti-occludin staining is predominantly detected at the cell borders (Fig. 5F), although there is still some cytoplasmic, off-target staining visible. Delivery of the two antibodies was studied with respect to the extracellular concentration required for sufficient loading and visualization (Fig. SI-1 of the supporting information). Even though antibodies of the same class should be similar in molecular size, shape and, thus, delivery efficiency, we found significant differences in the staining intensity. This finding may arise from the indirect detection using secondary antibodies that may have individual affinities to different primary antibodies.

It is a unique advantage of the small volume electroporation setup that it allows working with much smaller quantities of antibodies than other in situ electroporation devices to obtain the concentrations needed for intracellular delivery. Existing techniques and protocols require 0.2 to 10 ml of 5 µg/ml to 10 mg/ml antibody solutions^22,29–31^. These established devices are designed to load large numbers of cells needed for biomolecular detection methods such as western blotting or flow cytometry. The µISE-ECIS device only loads those cells residing on the electrode, but it allows monitoring these very same cells without any time delay. The non-invasive ECIS impedance measurements will report many potential effects induced by the specific binding of the antibodies. These effects include changes in cell junctions, cell morphology, or cell body dynamics as, for instance, during apoptosis. In addition, experiments can be designed to monitor the effects of antibody binding upon signal transduction cascades and other events that ultimately impact the cytoskeleton.

### Electrotransfection of adherent cells

Another important class of biomolecules capable of specifically modulating cell functions are encoding nucleic acids. Similar to antibodies, their consistent chemical nature provides many options to manipulate cellular properties once efficient loading protocols are established. Transfer of genetic material into cells with heterologous expression of recombinant genes is one of the most important tools in biomedical research. To date, electroporation is the simplest and most efficient *physical* transfection method that does not rely on chemical additives, potentially affecting cell viability^21,32^. Combining (1) the capability of electroporation of exogenous DNA into anchorage-dependent cells with (2) impedance-based sensing provides a novel tool to non-invasively monitor phenotypic changes induced by the expression of recombinant genes.

**Figure 6:**
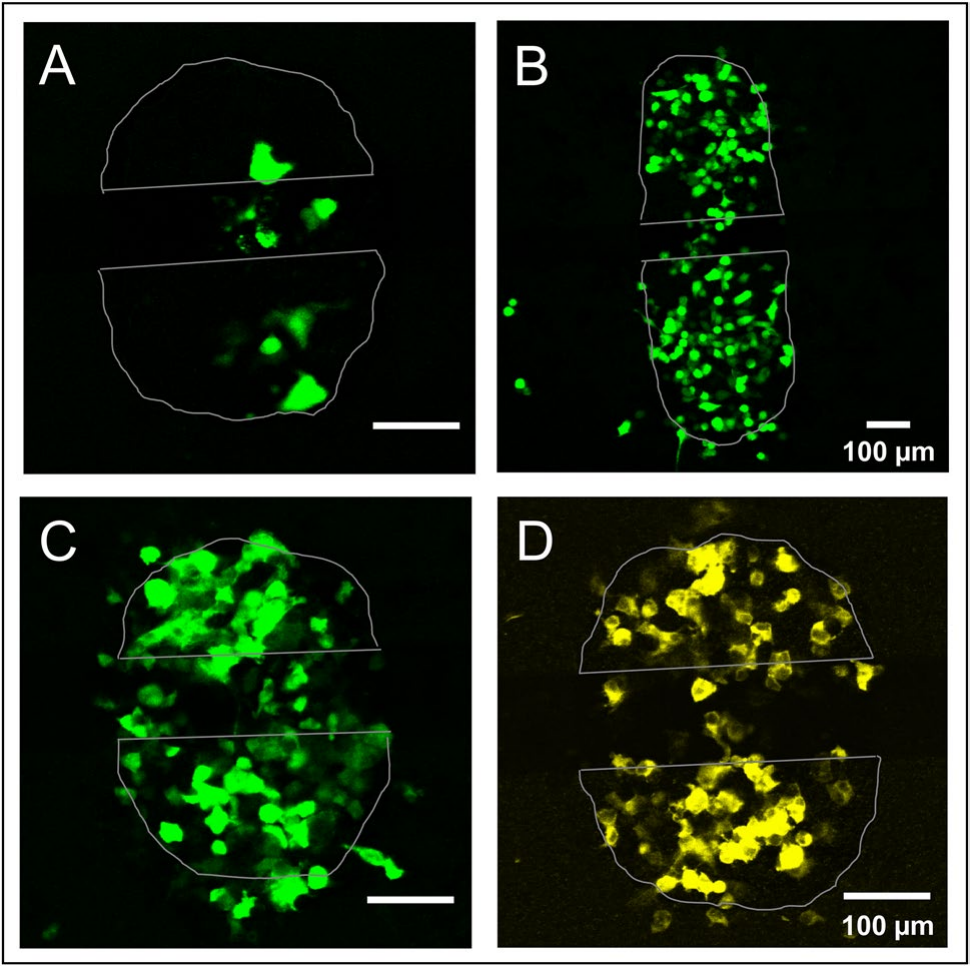
Electrotransfection of adherent mammalian cells with DNA expression plasmids. (**A**–**D**) Confocal fluorescence micrographs of adherent cells 24 h after electroporation in the presence of plasmid DNA encoding (**A**–**C**) EGFP (**A**: NRK; **B**: Hep-G2; **C**: HEK 293) or (**D**) EYFP-actin (HEK 293) The position of the electrodes are marked by grey outlines. In (**B**) a variant of small volume arrays with larger electrode areas (10^−3^ cm^2^) was used. Electroporation was performed with 200 ms electrical pulses of 5.0 V amplitude at an AC frequency of 40 kHz.

To demonstrate successful electrotransfection with the µISE-ECIS setup, the three cell lines NRK, HEK-293 and HEP-G2 were loaded with an expression plasmid encoding the green fluorescent protein (GFP). Figure 6 shows confocal fluorescence micrographs of the three cell lines 24 h after electrotransfection using in situ electroporation. The transfection efficiency is obviously dependent on cell type. Whereas HEK-293 and Hep G2 cells (Fig. 6B,C) show transfection efficiencies of about 70–80%, NRK cells were not very susceptible to electroporation-assisted transfection as revealed by only about 10% EGFP-positive cells on the electrodes (Fig. 6A). Transfected cells are not exclusively residing on the micro-electrodes but also in the non-conducting cleft between the electrodes (Fig. 6A–C). This is likely due to fringing of the electric field into the inter-electrode cleft, as it has been observed in dye loading studies for HEK-293 and Hep G2 cells as well (Fig. 3B,C). Fringing of the electric field may occur when the two opposing electrodes are close to each other and the field does not penetrate deeply into the bulk electrolyte. Under these conditions the electric field strength in the gap between the two electrodes is not zero and maybe sufficient for membrane permeabilization. An alternative explanation is cell migration and cell division within the 24 h between electroporation and microscopic inspection. The migration of transfected cells off the electrode was also found by other groups^33,34^. Results were not dependent on the nature of the expression plasmid as the chimeric actin-EYFP protein was also successfully expressed in HEK293 cells after electrotransfection (Fig. 6D).

Compared to viral transduction, the observed expression of the reporter genes is rather modest. However, similar transfection efficiencies have also been reported for other in situ electroporation approaches using coplanar arrangements of film-electrodes^33,35^. In contrast to the very efficient delivery of small dye molecules (cp. Figure 3) via diffusion through transient pores in the membrane, genetic material is transferred into the cytosol by mechanisms not fully understood yet. The current understanding of the process implies an electrophoretic collection of DNA molecules atop the membrane that occurs concomitantly with membrane destabilization by the electric field as the basis for membrane-mediated uptake^36^. Accordingly, the transfection efficiency observed for different cell types depends progressively upon the specific molecular architecture of the membrane, the cells’ mitotic activity at the time of electrotransfection, and the individual activity of the transcription and translation machinery. The restricted accessibility of DNA to the nucleus in confluent cell layers is probably the major bottleneck for high transfection efficiencies, as the nuclear envelope is not permeabilized by the electroporation pulses (cp. Figure 3). Only during mitosis, when the nuclear envelope is decomposed, are large DNA molecules able to move from the cytoplasm to the nucleus. This is typically not the case for the confluent cell layers used in the experiments described here. Cell type-specific transfection efficiencies for NRK, HEK-293, and Hep G2 cells might therefore depend largely on the ability to have mitosis in the cell layer. Hence, it seems straightforward to perform electrotransfection with subconfluent, sparse cell layers. But a subconfluent coverage of the electrode provides sneak pathways for the current such that the electrical pulses quickly lose their effectiveness for electro-permeabilization. The current limitations of DNA transfection may be overcome by using RNA instead of DNA for cell manipulation, as RNA does not need to migrate to the nucleus to become biologically active.

### Electroporation in the presence of enzymes and bioactive proteins

**Figure 7:**
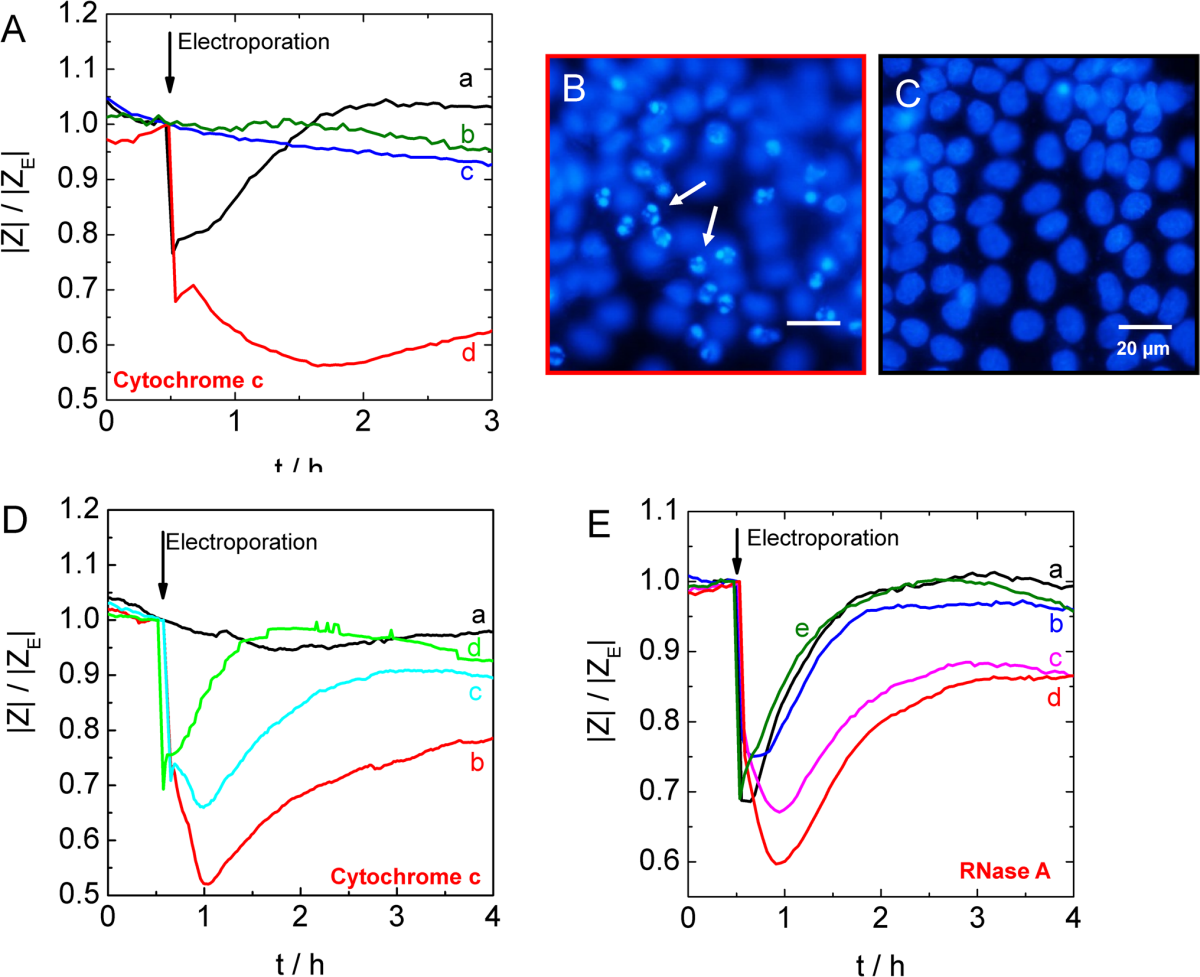
Cellular response after electroporation-mediated loading of NRK cells with small proteins and enzymes. (**A**) Time course of the normalized impedance |Z|/|Z_E_| upon electroporation (arrow) of NRK cells in presence of 10 mg/ml cytochrome c (d: red line). Control cells were incubated with cytochrome c but not exposed to electroporation (c: blue line) or exposed to electroporation in the absence of cytochrome c (a: black line) or remained entirely untreated (b: green line). (**B**, **C**) Epifluorescence micrographs of DAPI-stained NRK cells grown on microelectrodes approximately 3 h after electroporation with (**B**) or without (**C**) 10 mg/ml cytochrome c in the incubation buffer. (**D**) Time course of the normalized impedance |Z|/|Z_E_| upon electroporation (arrow) of NRK cells in the presence of 10 mg/ml cytochrome c (b: red line), in co-presence of 10 mg/ml cytochrome c and 150 µM of the caspase inhibitor peptide DEVD (c: cyan line), or upon co-electroporation with 10 mg/ml cytochrome c and 250 µM caspase inhibitor (d: green line). Control cells were incubated with 10 mg/ml cytochrome c and 250 µM caspase inhibitor but not exposed to electroporation (a: black line). (**E**) Time course of the normalized impedance |Z|/|Z_E_| upon electroporation (arrow) of NRK cells in presence of RNase A. The concentrations of RNase A were 0 mg/ml (a: black line), 0.1 mg/ml (b: blue line), 0.25 mg/ml (c: magenta line) and 0.5 mg/ml (d: red line). Heat inactivated RNase (0.25 mg/ml) (e: green line). Electroporation was performed with 200 ms electrical pulses of 5.0 V amplitude at an AC frequency of 40 kHz.

In preceding experiments, we demonstrated the successful loading of cells with various biomolecules and experimental probes. Here we demonstrate that the response of NRK cell layers to the electroporation-mediated injection of proteins with cytoplasmic activity is readily monitored by time-resolved impedance readings. Cytochrome C and RNase A were used as model proteins that were introduced into the cytosol by electroporation. Cytochrome c is a protein of the electron transport chain (ETC) that is bound to the inner mitochondrial membrane under resting conditions. When the mitochondria become dysfunctional, the protein is released to the cytosol, and it triggers and amplifies programmed cell death (apoptosis) by activation of the apoptosome and downstream effector caspases^37^. Apoptotic cells condensate their chromatin, dismantle cellular components, shrink, disintegrate from the cell layer and finally detach from their substrate. In the experiment presented in Fig. 7A, a confluent layer of NRK cells was electroporated with cytochrome c at a concentration of 10 mg/ml in the extracellular buffer. In the presence of cytochrome c, cell recovery after the electric pulse (arrow) is significantly delayed (curve d) compared to electroporation in the absence of cytochrome c (curve a). Moreover, a cell layer that was incubated with 10 mg/ml cytochrome c, but not subjected to electroporation, did not show any measurable response within the first three hours (curve c) relative to an untreated control (curve b). About 2.5 h after electroporation in the presence of cytochrome c, the impedance value is still close to 60% of the pre-pulse impedance, while control cell layers subjected to electroporation in cytochrome-free buffer completely recovered from the electric pulse within one hour. The transient drop of the normalized impedance after pulse application and its recovery within approximately 60 min (curve a) reflects the typical response profile of NRK cell layers to electroporation in buffer^12^. This response is due to pulse-induced changes in cell shape rather than the short-lived membrane permeabilization itself^12^.

The onset of apoptosis induced by the presence of cytochrome c in the cytoplasm is likely responsible for the persistent impedance decrease over several hours. This data interpretation is supported by microscopic studies after labeling the DNA with DAPI. Approximately 3 h after electroporation in the presence of cytochrome c, NRK cells show clear indications for chromatin condensation (Fig. 7B), whereas the nuclei are unaffected when electroporation is performed in the absence of cytochrome c (Fig. 7C). For a more specific proof of this mechanism of action, we co-electroporated confluent NRK cells with 10 mg/ml cytochrome c and increasing concentrations of the modified tetrapeptide Ac-DEVD-CHO which is a well-known inhibitor of caspases^38^. The latter catalyze the self-digestion of cell components during apoptosis. Curve c in Fig. 7D shows the response of NRK cells to injection of cytochrome c when 150 µM of this peptide is present during electroporation as well. Trace d represents the corresponding response profile in the presence of 250 µM Ac-DEVD-CHO. It is evident that the co-presence of the caspase inhibitor in the cytoplasm reduces the impact of cytochrome c. For the highest concentration, the impedance time course closely resembles control conditions. Please note that the time courses in Fig. 7A(d) and 7D(b) represent the same exposure to cytochrome c but have been recorded with cells from different batches and in different passage numbers several months apart. It is not surprising that manipulating intracellular signaling cascades depends critically on cell status.

Figure 7E presents the response of NRK cells to electroporation in the presence of different concentrations of the RNA degrading ribonuclease RNase A. RNases cleave cellular mRNA and tRNA molecules which are essential molecular species in protein synthesis^39^. Therefore, it was expected that cytoplasmic delivery of the enzyme might inhibit essential cell functions. Microinjection of RNase A in Xenopus oocytes was shown to abolish protein synthesis at concentrations of only 0.03 nM^40^. Inhibition of protein synthesis is a typical intracellular stress signal that is assumed to induce apoptosis by yet unknown pathways^41^. Interestingly, due to their cytotoxic potential, ribonucleases have been proposed as drug candidates in chemotherapy^42^. In most studies presented to date, however, RNases have been exclusively applied extracellularly and, thus, their intracellular activity was dependent upon cellular uptake mechanisms such as endocytosis^43^. Glogauer and McCulloch electroporated suspended fibroblasts in the presence of bovine pancreatic RNase A^44^. They detected a concomitant reduction of cytoplasmatic RNA by Pyronin Y staining of RNA and subsequent flow cytometry analysis, but did not specifically quantify any further effect of RNA reduction on cell viability. Figure 7E summarizes the impedimetric response of confluent NRK cells upon electroporation-mediated injection of increasing concentrations of RNase A. Under control conditions, NRK cells were electroporated in the absence of RNase (curve a). The lowest concentration of RNase A (0.1 mg/ml) showed no significant effect on cell recovery after electroporation (curve b) compared with control. When the extracellular concentration of RNase was increased to 0.25 mg/ml (curve c) or 0.5 mg/ml (curve d), recovery to the pre-pulse impedance was considerably delayed but incomplete, as impedance values returned to only 85% of the pre-pulse values by three hours after electroporation. When the experiment was repeated with the same amount of heat-inactivated RNase A (curve e), the impedance response after electroporation was essentially that of the control (curve a). This experiment demonstrates that only the injection of biologically active proteins into the cytoplasm induces a significant cell response. Solely extracellular application of RNase A was found to be non-toxic up to 100 μM by others^45^, a level approximately 3 times higher than the highest concentration used in this work (0.5 mg/ml:~ 36 μM). The surprisingly short-lived effect of the RNase A injection is very likely due to intracellular inhibition of RNase A by ubiquitous cellular RNase inhibitors^46^. Moreover, it is noteworthy that the µISE-ECIS approach will only affect those cells that reside on the electrode experiencing field-induced membrane permeabilization. All cells residing off the electrode are not manipulated. Thus, when the cells on the electrode die, live cells from the periphery may migrate into the open spaces and repopulate the electrode. Accordingly, the impedance may be observed to only transiently approach cell-free values. This phenomenon has been described before and is the technical basis for an ECIS-based wound healing assay^47^.

The data presented in Fig. 7 illustrates that the μISE-ECIS approach enables rather complex intracellular manipulations by introducing a variety of membrane-impermeable compounds into the cytoplasm while the cell response is monitored in real-time.

## Conclusion

The miniaturized setup for combined in situ electroporation and impedance monitoring of adherent cells (µISE-ECIS) provides an unprecedented experimental option. With this approach, one can introduce biologically active, but membrane-impermeable molecules such as enzymes, antibodies or encoding nucleic acids into the cytoplasm and then follow the cell response using real-time impedance measurements. This paves the way to specifically manipulate intracellular signalling cascades, metabolic pathways, or mechanistically important protein–protein interactions and read the associated consequences for certain cell functions or phenotypes. Due to the quick recovery of the cells from the electroporation pulse, the lag time between efficiently loading and reliably observing cells is reduced to 45 to 60 min—much more rapidly when compared to manipulating anchorage-dependent cells in suspension. The small volume required to perform the assay allows for affordable and systematic studies, even with very precious and expensive biomolecules. The experimental option to introduce nanoparticles, in particular those carrying sensing capabilities, into the cytoplasm of adherent cells may also become a disruptive new methodology combining intracellular chemosensing with holistic cell analysis. As all operations are electrically in nature, miniaturized, and computer-controlled, the µISE-ECIS approach could easily be integrated into organ-on-chip or other microfluidic cell culture devices.

## Supplementary information

Supplementary information.
