# Supplementary material for: Impedance analysis of adherent cells after in situ electroporation-mediated delivery of bioactive proteins, DNA and nanoparticles in µL-volumes
Source: Sci Rep. 2020 Dec 7;10:21331. doi: 10.1038/s41598-020-78096-6 (PMC7721805; doi:10.1038/s41598-020-78096-6)
Supplement: Supplementary file 1 — Supplementary information. [file 41598_2020_78096_MOESM1_ESM.docx]

**Supporting Information**

**Impedance Analysis of Adherent Cells after *in situ* Electroporation-Mediated Delivery of Bioactive Proteins, DNA and Nanoparticles in µL-Volumes**

Judith A. Stolwijk^1*^ and Joachim Wegener^1,2*^

^1^ Institut fuer Analytische Chemie, Chemo- & Biosensorik, Universität Regensburg, Universitaetsstr. 31, 93053 Regensburg (G)

^2^ Fraunhofer Einrichtung fuer Mikrosysteme und Festkörpertechnologien EMFT, Universitaetsstr. 31, 93053 Regensburg (G)

*Corresponding Authors

Prof. Dr. Joachim Wegener

Tel.: +49-941-943-4546

Fax: +49-941-943-4491

Email: [Joachim.Wegener@ur.de](mailto:Joachim.Wegener@ur.de)

Dr. Judith Anthea Stolwijk

Tel.: +49-941-943-4048

Email: Judith.Stolwijk@ur.de

**Electroporation-mediated loading of adherent cells with antibodies: concentration dependence**

Electroporation mediated delivery of the two primary antibodies *anti-β-catenin* and *anti-occludin* was also studied as a function of their extracellular concentration during electroporation. The corresponding fluorescence micrographs are summarized in figure SI-1A for *anti-β-catenin* and in figure SI-1B for *anti-occludin*. The µISE-experiments and the work-up of the samples were performed as described in the main manuscript. Even though both primary antibodies have been raised in the same species (rabbit), were of the same type (IgG) and were visualized with the identical secondary antibody, we observed very different staining intensities for similar concentrations. The reasons for this are unclear but are very likely not a consequence of the electroporation-mediated delivery as we observed the same phenomenon for conventional immunostaining. Even here, the concentrations required for decent staining were different by a factor of 6. The conventional immunostaining was performed as described in the materials and methods section of the main manuscript.


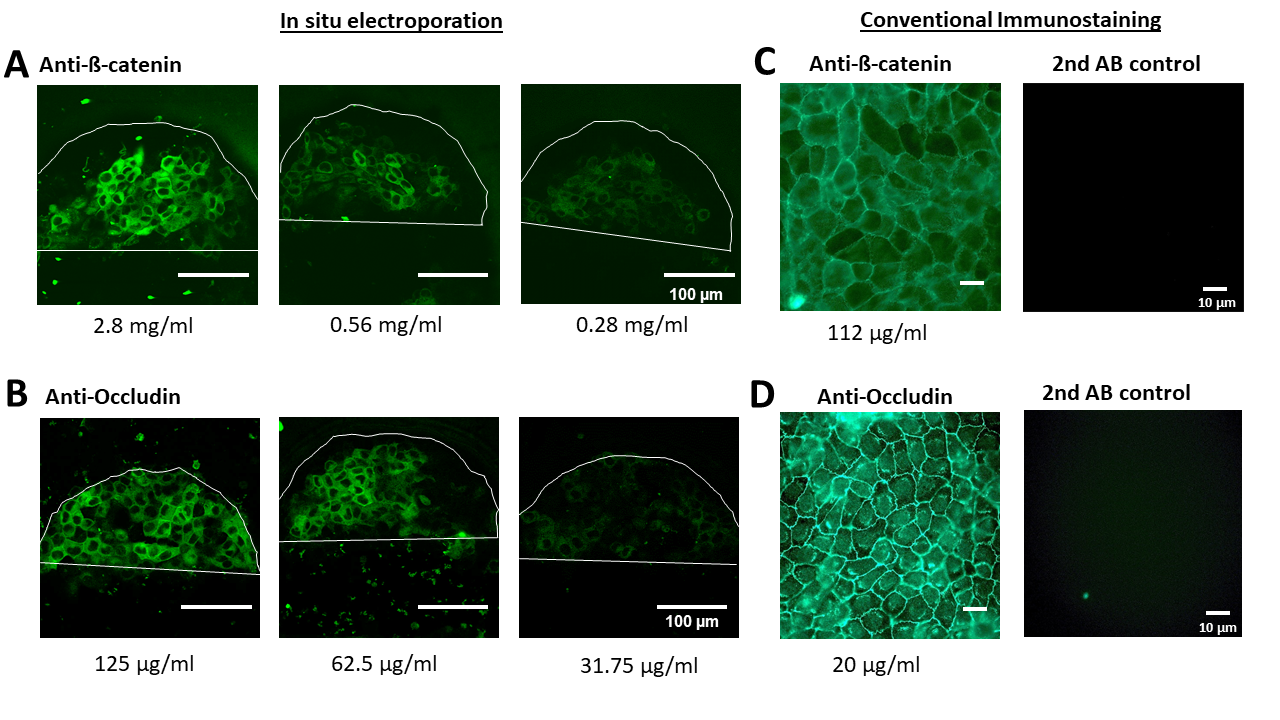


**Figure SI-1.** µISE-mediated loading of NRK cells with antibodies at different concentrations. **(A, B)** Confocal fluorescence micrographs of NRK cells grown on half-circular electrodes (marked by grey outlines) after electroporation in the presence of an **(A)** anti-ß-catenin antibody (at concentrations of 2.8, 0.56, or 0.28 mg/mL) or **(B)** anti-occludin antibody (at concentrations of 125, 62.5, 31.75 µg/mL) followed by fixation, permeabilization and labeling of the bound primary antibody with an Alexa Fluor^®^ 488-labeled secondary antibody. Electroporation was performed with 200 ms electrical pulses of 5.0 V amplitude at an AC frequency of 40 kHz. **(C, D)** Wide-field fluorescence micrographs of NRK cells grown on glass slides subjected to a conventional immunostaining with **(C)** anti-ß-catenin antibody (at a concentration of 112 µg/mL) or **(D)** anti-occludin antibody (at a concentration of 20 µg/mL), including respective secondary antibody controls (2nd AB control).
